# College and Hospital Notes

**Published:** 1906-12

**Authors:** 


### COLLEGE AND HOSPITAL NOTES.

The Buffalo Homeopathic Hospital is in need of greater accommodations and increased facilities for work. For years the staff of physicians and nurses have struggled under the most serious lack of equipment and accommodations, with results that thoroughly testify to their skill and devotion. Now, however, plans have been formulated that will require $100,000 and call for the sale of the present hospital at Cottage and Maryland streets, the purchase of a new site and the erection of a modern hospital building, in some place where the requisite quiet can be secured, with, at the same time, proper street car communication.

Up to date $65,000 have been subscribed and the remaining $35,000 will, it is hoped, be forthcoming within a short time.

Subscriptions may be addressed to H. E. Montgomery, Court and Wilkenson streets. Subscription blanks can also be obtained of Dr. George T. Moseley, chairman of the building fund committee, 202 Delaware avenue.

Dr. L. Duncan Bulkley is giving the eighth series of Clinical Lectures on Diseases of the Skin, in the out-patient hall of the New York Skin and Cancer Hospital, which commenced Wednesday afternoon, November 7th, 1906, at 4:15 o'clock. The course is free to the medical profession.

The Frontier Hospital is the name given for a new hospital which is proposed to be established at the corner of Main and Riley streets, Buffalo. Several business men are promoting the scheme and it is intended to organise and incorporate a company. According to plans published in the newspapers the hospital will be conducted on the club order, each member paying two dollars for a certificate entitling the holder, in case of serious injury or disability, to ambulance service, all surgical treatment, including bandages, dressing, medicine, bed, board and nursing until recovery, for one year from date of the issuing of the certificate. For diseases not arising from accidents, a nominal charge of $1 a day will be made for bed, board and attendances.

Family certificates, costing $5, will give husband, wife and children of the family under 16 years old, the same privileges for a period of one year.

